# Complex free-space magnetic field textures induced by three-dimensional magnetic nanostructures

**DOI:** 10.1038/s41565-021-01027-7

**Published:** 2021-12-20

**Authors:** Claire Donnelly, Aurelio Hierro-Rodríguez, Claas Abert, Katharina Witte, Luka Skoric, Dédalo Sanz-Hernández, Simone Finizio, Fanfan Meng, Stephen McVitie, Jörg Raabe, Dieter Suess, Russell Cowburn, Amalio Fernández-Pacheco

**Affiliations:** 1 Cavendish Laboratory, University of Cambridge, Cambridge, UK; 2 Max Planck Institute for Chemical Physics of Solids, Dresden, Germany; 3 SUPA, School of Physics and Astronomy, University of Glasgow, Glasgow, UK; 4 Departamento de Física, Universidad de Oviedo, Oviedo, Spain; 5 CINN (CSIC–Universidad de Oviedo), El Entrego, Spain; 6 University of Vienna Research Platform MMM Mathematics–Magnetism–Materials, Vienna, Austria; 7 Swiss Light Source, Paul Scherrer Institute, Villigen, Switzerland; 8 Berlin Partner für Wirtschaft und Technologie GmbH, Berlin, Germany; 9 Instituto de Nanociencia y Materiales de Aragón, CSIC-Universidad de Zaragoza, Zaragoza, Spain

**Keywords:** Magnetic properties and materials, Spintronics, Magnetic properties and materials, Nanowires, Magnetic properties and materials

## Abstract

The design of complex, competing effects in magnetic systems—be it via the introduction of nonlinear interactions^1–4^, or the patterning of three-dimensional geometries^5,6^—is an emerging route to achieve new functionalities. In particular, through the design of three-dimensional geometries and curvature, intrastructure properties such as anisotropy and chirality, both geometry-induced and intrinsic, can be directly controlled, leading to a host of new physics and functionalities, such as three-dimensional chiral spin states^7^, ultrafast chiral domain wall dynamics^8–10^ and spin textures with new spin topologies^7,11^. Here, we advance beyond the control of intrastructure properties in three dimensions and tailor the magnetostatic coupling of neighbouring magnetic structures, an interstructure property that allows us to generate complex textures in the magnetic stray field. For this, we harness direct write nanofabrication techniques, creating intertwined nanomagnetic cobalt double helices, where curvature, torsion, chirality and magnetic coupling are jointly exploited. By reconstructing the three-dimensional vectorial magnetic state of the double helices with soft-X-ray magnetic laminography^12,13^, we identify the presence of a regular array of highly coupled locked domain wall pairs in neighbouring helices. Micromagnetic simulations reveal that the magnetization configuration leads to the formation of an array of complex textures in the magnetic induction, consisting of vortices in the magnetization and antivortices in free space, which together form an effective *B* field cross-tie wall^14^. The design and creation of complex three-dimensional magnetic field nanotextures opens new possibilities for smart materials^15^, unconventional computing^2,16^, particle trapping^17,18^ and magnetic imaging^19^.

## Abstract (web summary)

In magnetic double nanohelices, the balance of geometrical effects and dipolar interaction results in strongly coupled three-dimensional spin states. This leads to topological features in the stray field, offering a new route to pattern the magnetic induction.

## Main

**Fig. 1:**
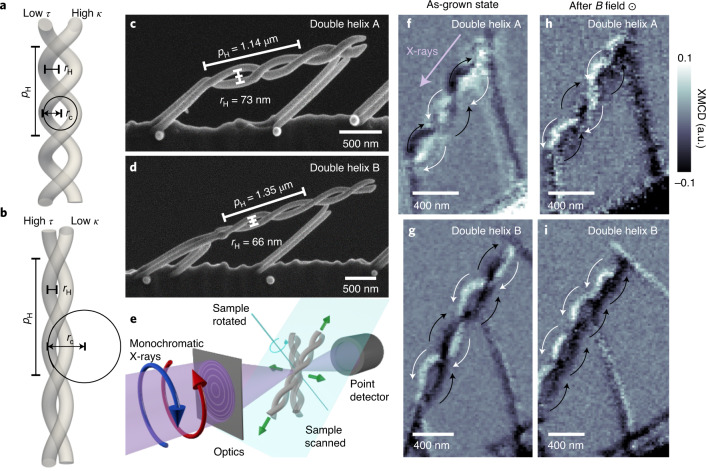
Ferromagnetic double helices. **a**,**b**, The pitch *p*_H_ and radius *r*_H_ of the helix determine the radius of curvature *r*_c_ = 1/*κ* and the torsion *τ* of the system. **c**,**d**, Ferromagnetic double-helix nanostructures, with varying *p*_H_ and *r*_H_ (2*r*_H_ indicated) and a nanowire diameter of ~70–80 nm. Additional straight cobalt pillars are included to sustain the nanostructure and facilitate the X-ray microscopy experiments. **e**, The magnetic state is probed using STXM and XMCD using a laminography set-up for three-dimensional imaging, providing nanoscale projections of the magnetization parallel to the X-ray direction (indicated by the purple arrow in **f**). **f**,**g**, In the as-grown state both double helices are composed of two fully black and white helices—corresponding to antiparallel-magnetized single-domain helices. **h**,**i**, After the application of a saturating field transverse to the helix long axis, double helix A returns to the antiparallel state (**h**), while double helix B remains in a multidomain state identified by alternating bright and dark regions within the individual helices (**i**). Black and white arrows indicate the direction of the magnetization in each image. a.u., arbitrary units.

We consider a model system that consists of two intertwined, yet spatially separated, ferromagnetic nanohelices. This three-dimensional nanomagnetic system has a complex energy landscape defined by the balance of competing intra- and interhelix effects (terms defined above). The nanoscale double helix combines effects of curvature and torsion that may result in curvature-induced magnetic anisotropy and chirality effects^20–22^. Specifically, the two helices are designed to have the same chirality, and are offset by half a period, leading to a constant interhelix separation along the length of the system. We fabricate the system of two intertwined cobalt nanohelices with focused electron beam induced deposition^23^. Scanning electron microscope (SEM) images of two nanoscale double helices are presented in Fig. 1: the first (double helix A, Fig. 1c) with lower pitch and higher radius, the second (double helix B, Fig. 1d) more elongated with higher pitch and lower radius (geometries defined in Table 1). Both double helices have a nanowire diameter of approximately 70–80 nm with an interhelix distance of ~50–70 nm and therefore exhibit strong magnetostatic coupling.

**Table 1:** Parameters of the three-dimensional geometries of the two nanoscale double helices investigated in this work and shown in Fig. 1c,d

	Nanowire diameter	Helix radius, *r*_H_	Helix pitch, *p*_H_	Radius of curvature, *κ*^−1^	Radius of torsion, *τ*^−1^
Helix A	80 nm	73 nm	1.14 μm	523 nm	210 nm
Helix B	80–85 nm	66 nm	1.35 μm	765 nm	235 nm

To probe the magnetic state of these complex three-dimensional magnetic nanostructures, we employ scanning transmission X-ray microscopy (STXM, Fig. 1e). By tuning the X-ray energy to the Co L_2_ edge (796 eV) and measuring images with circular polarization, we exploit X-ray magnetic circular dichroism (XMCD) to obtain a high-spatial-resolution projection of the magnetization parallel to the X-ray beam (Fig. 1f). We first probe the as-grown state of the two magnetic double helices in Fig. 1f,g, where we can see that in both XMCD images the double helices are composed of a dark and a bright helix, which corresponds to the individual helices being in antiparallel-magnetized single-domain states with a quasi-tangential magnetization distribution. This is expected due to the radii of curvature and torsion (defined in Table 1) being much larger than both the exchange length (4–6 nm) and the diameter of the nanowires^20^. These two antiparallel double-helix states (magnetizations of helices A and B either positive and negative or negative and positive, respectively) represent the degenerate ground states of the system (Methods), and are a result of the fabrication sequence of the helices, which are grown in parallel: at the start of the growth when the helices are small, the magnetic moments reorient to minimize the magnetostatic energy, aligning antiparallel to one another. This antiparallel state is maintained as the helices are grown, leading to the formation of these single-domain, micrometre-length structures^7^.

Although the two double-helix systems form similar antiparallel states in their as-grown configuration, they exhibit very different configurations following the application of a magnetic field perpendicular to the long axis of the helix. The XMCD projection of double helix A again reveals a pair of dark and bright helices, indicating the return to an antiparallel state (specifically the opposite antiparallel state, Fig. 1h). However, the XMCD projection of double helix B is different, with alternating regions of dark and bright contrast within individual helices (Fig. 1i), indicating the formation of a multidomain state with a regular array of domain walls. With both double-helix systems composed of the same material and exposed to the same external magnetic field, we attribute this difference in behaviour to their different curvatures, torsions and interhelix couplings.

**Fig. 2:**
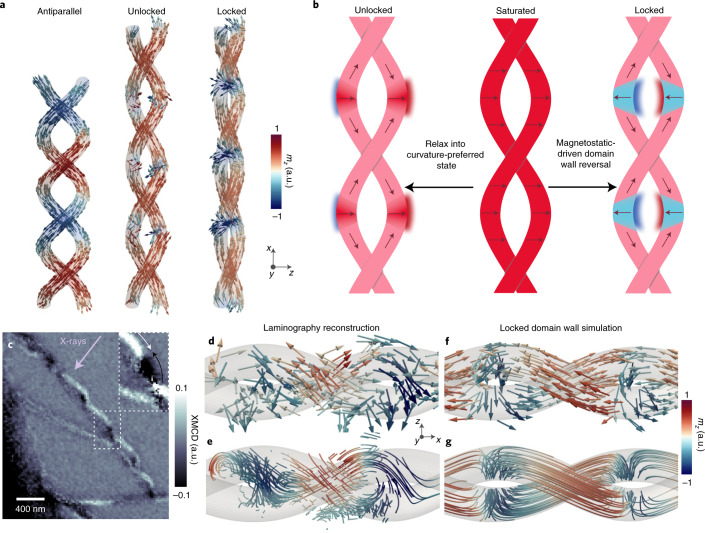
The ‘locked’ domain wall state. **a**, Finite-element micromagnetic simulations of double helices with varying pitch and radius reveal three stable configurations after the presence of a transverse saturating magnetic field: the antiparallel state, as well as regularly spaced unlocked and locked domain wall pairs. **b**, When the locked state is the stable remanent state, the saturated state relaxes to the unlocked domain wall state, before the domain wall pairs reorient due to the magnetostatic interaction to form the locked state. **c**, An XMCD projection of double helix B rotated by 60° from Fig. 1 reveals a periodic array of domain walls (with the position of one domain wall revealed by the transition from black to white and indicated by arrows in the inset). X-ray direction indicated by purple arrow. **d**,**e**, Soft-X-ray laminography reveals the three-dimensional structure of the domain walls, with the reconstructed magnetization represented by arrows (**d**) and streamlines (**e**), revealing a figure-of-eight texture. **f**,**g**, The presence of the locked domain wall state is confirmed by comparison with micromagnetic simulations (**f**), with streamlines indicating the direction of the magnetization (**g**) again revealing the recognizable figure-of-eight texture in the magnetization that indicates the reversal of the domain walls.

To elucidate the influence of the three-dimensional geometry on the remanent magnetic configuration, we simulate the magnetic configuration formed after the application of a saturating transverse magnetic field for a variety of helix pitches and radii using finite-element micromagnetic simulations^24^. We identify three remanent magnetic configurations. The first is the antiparallel state (Fig. 2a, left), as observed experimentally for double helix A (Fig. 1f). The second is an unlocked domain wall state (Fig. 2a, centre), in which the transverse domain walls are aligned in the direction of the applied magnetic field. This unidirectional state is characterized by having the net magnetic surface charge of the walls located at the outer curved section of the wires^25,26^, as favoured by the curvature-induced anisotropy and the curvature-induced Dzyaloshinskii–Moriya interaction (DMI), which promote a particular domain wall chirality^25^. This state is consistent with the equivalent magnetic configurations of planar magnetic nanowires^27^. For geometries that host the unlocked state at remanence, the curvature-induced effects dominate over the interstructure magnetostatic interaction. We also observe a third, unconventional domain wall configuration (Fig. 2a, right), in which the domain walls fully reverse with respect to both the direction of the applied magnetic field and the curvature-induced DMI, becoming locked in place due to the strong interhelix interaction, as shown schematically in Fig. 2b.

**Extended Data Fig. 1:**
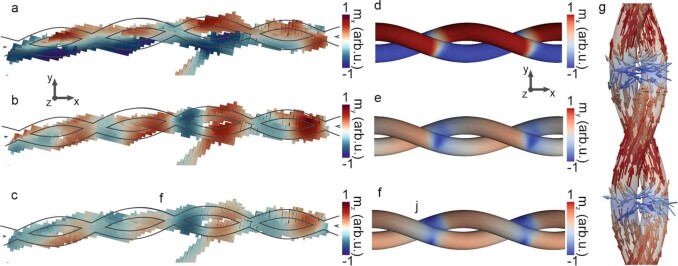
Comparison of experimental reconstruction with simulations. The reconstructed x, y and z components of the magnetisation through a centre slice of the helix (a–c, respectively, with outlines indicating the helix structure) compared with the corresponding components of the magnetisation (d–f) of the micromagnetic simulation shown in g.

To determine whether the locked domain wall state is present in double helix B, we perform soft-X-ray magnetic laminography^12,13,28,29^ (Fig. 1e) to map its three-dimensional magnetization vector field with nanoscale resolution The reconstructed magnetization is given by arrows in Fig. 2d, where a reversal of the direction of the magnetization within the magnetic domain walls can be observed, consistent with the locked domain wall state. An additional representation of the magnetization with streamlines (Fig. 2e) reveals a distinctive figure-of-eight structure in the reconstructed magnetization. When compared with micromagnetic simulations in Fig. 2f,g (see also Extended Data Fig. 1), the figure-of-eight structure is reproduced, providing confirmation of the reversal of the domain wall direction and the resulting locked domain wall array in double helix B.

The formation of these different remanent states—the antiparallel state and locked domain wall state for double helices A and B, respectively—occurs due to the geometry of the double helices strongly affecting the competing interactions. Specifically, the higher torsion:curvature ratio of double helix B, associated with its higher helix pitch and lower helix radius, promotes the formation of a stable array of locked domain walls. After the formation of the unlocked domain walls following transverse saturation, the domain walls reorient owing to the magnetostatic interaction overcoming the curvature-induced DMI of the domain wall. In particular, the higher torsion:curvature ratio decreases the distance between the helices, increasing the magnetostatic interaction between them, while at the same time decreasing the curvature-induced DMI, promoting the rotation of the domain walls to the locked state (Methods). This reorientation creates a more confined magnetic flux, reducing the magnetostatic energy, as shown schematically in Fig. 2b. In contrast, the antiparallel state observed in double helix A forms due to the higher curvature and lower torsion. Specifically, as the interdomain wall distance in different helices increases, the coupling between domain walls in different helices decreases, and the distance between domain walls within a single helix is reduced. Both effects favour the annihilation of neighbouring domain wall pairs and the formation of the antiparallel state. This geometry-dependent behaviour in intertwined double helices is confirmed both by mapping the phase diagram of this system with micromagnetic simulations and by an analytical model (Methods and Supporting Sections I and II), confirming that the locked domain wall state forms as a result of the influence of the geometry on these competing effects.

**Fig. 3:**
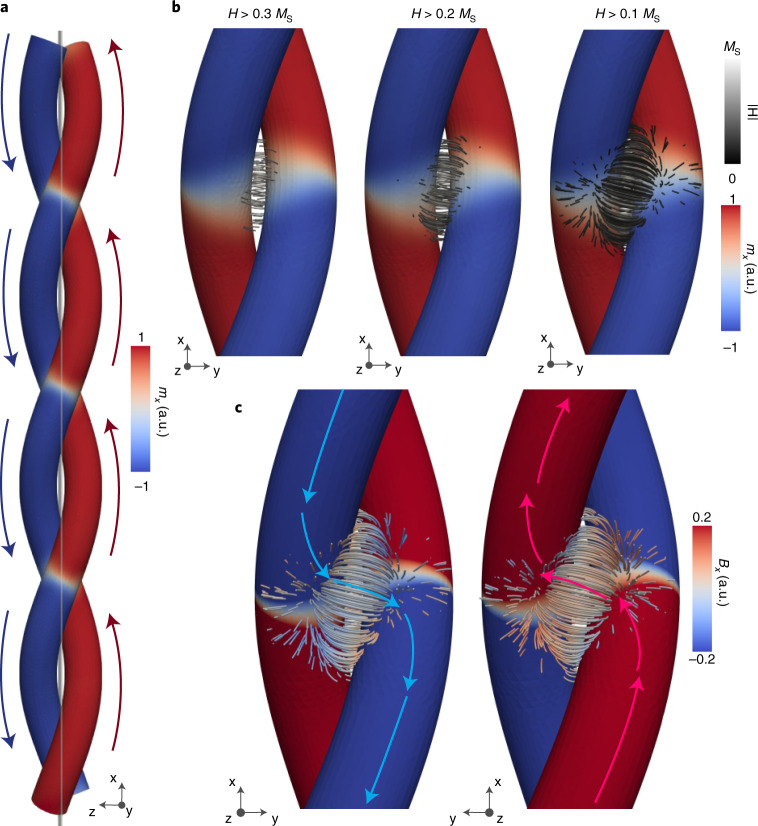
Flux channelling in the locked domain wall state. **a**, The array of domain walls leads to the double helix being split into negative (left, blue) and positive (right, red) *m*_*x*_ domains. **b**, **H** of a locked domain wall pair is plotted, with its magnitude indicated by the black–white colour scale, indicating that, while the strongest fields (*H* > 0.3 *M*_S_) align horizontally between domain walls, weaker but still significant components of the stray field (*H* > 0.2 *M*_S_ and *H* > 0.1 *M*_S_) rotate into the *x* direction, forming a ‘flux channel’ between magnetic domains of the same axial direction. **c**, This flux channel is confirmed by plotting the direction of the stray field with the magnetization on either side of the double helix, where the stray field connects magnetic domains of the same direction, but in different helices (indicated by blue and red arrows).

The locked domain wall state observed here occurs due to the balance between intrahelix properties and interhelix coupling. While it is known that curvature and torsion influence intrananowire properties such as anisotropies and chirality^5,20–22,30^, their influence on internanostructure coupling—that is, the magnetic stray field generated by neighbouring three-dimensional structures—remains unexplored. To elucidate the influence of the three-dimensional geometry on the magnetostatic coupling, we calculate the magnetic induction **B** = *μ*_0_(**H** + **M**) in the whole space (including both the magnetic material and free space) by taking the magnetization configuration **M** of the locked domain wall state from micromagnetic simulations and computing its stray field (**H**). We first consider the overall structure of **B** within the helix: the formation of the regular array of domain walls results in the double helix being split into two main domains, as shown in Fig. 3a, where on the left the magnetization points down along $- \hat {\mathbf{x}}$ (blue), and on the right the magnetization points up along $+ \hat {\mathbf{x}}$ (red). This asymmetry in **B** within the double helix results in a similar asymmetry in **B** in free space, seen by considering the variation in the stray field surrounding the domain walls. While the highest magnitude stray fields in free space (${{{\mathit{B}}}} = \mu _0{{{\mathit{H}}}} > 0.3\mu _0M_{\mathrm{s}}$) are found to mostly align horizontally ($\hat {\mathbf{y}}$) between the domain wall pairs (left panel of Fig. 3b), as lower-magnitude stray fields are considered (middle and right panels of Fig. 3b) we observe a growing component of the stray field in the plane of the domain wall cross-section (*x*–*z* plane), which becomes more noticeable when weaker stray fields of magnitude >0.1 *M*_s_ are plotted. In fact, the stray field is seen to rotate asymmetrically into the *x*–*z* plane to channel the magnetic flux of magnetic domains of the same direction in the two different helices (Fig. 3c): on one side the stray field develops a (blue) negative vertical $\hat {\mathbf{x}}$ component to channel the flux of the (blue) negative *m*_*x*_ domains, while on the other side the stray field tilts into the (red) positive $\hat {\mathbf{x}}$ direction to connect domains of (red) positive *m*_*x*_, indicated by blue and red arrows in Fig. 3c.

**Fig. 4:**
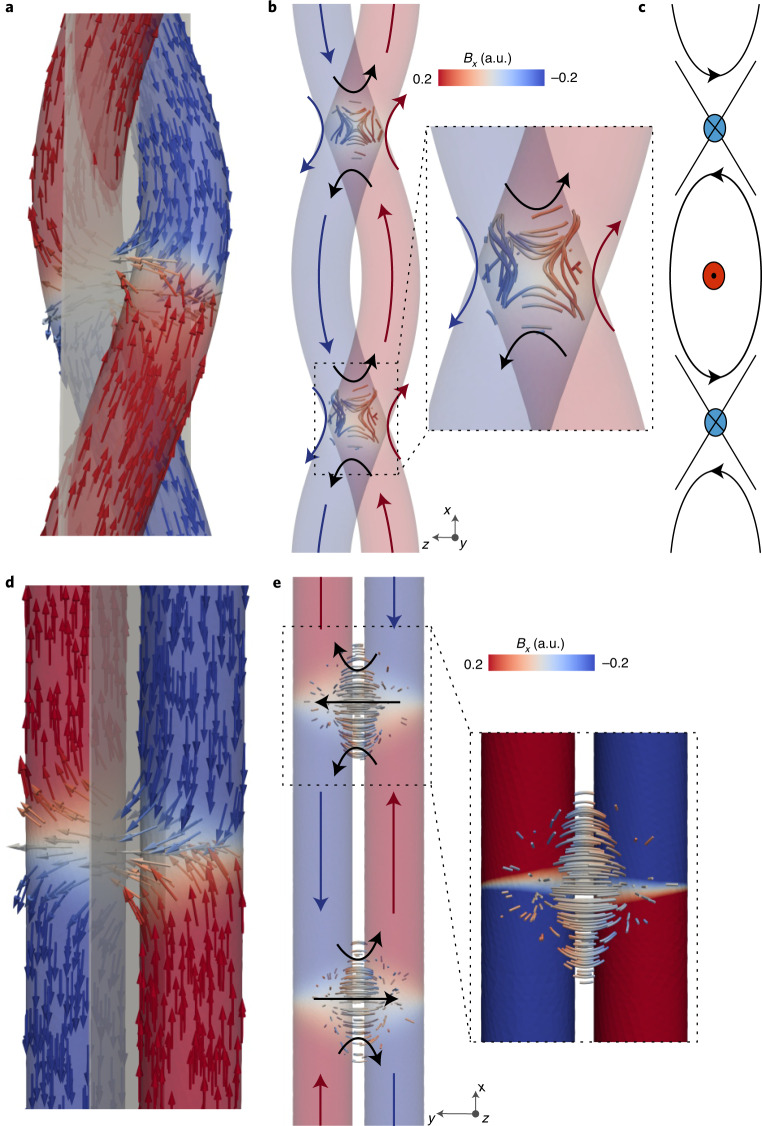
Textures in the **B** field of the locked domain wall state. **a**,**b**, Plotting the induction in the *x*–*z* plane (plane indicated with respect to magnetic configuration in **a**), an array of antivortices in the in-plane components of the **B** field, plotted with streamlines, is observed (**b**), in between effective vortices formed by the chiral magnetization. The structure of the antivortex is shown in greater detail in the inset. Schematic arrows are added to show the direction of **B**. **c**, The array of vortices and antivortices resembles the structure of a cross-tie domain wall. **d**,**e**, These textures in the stray field do not occur in the corresponding configuration of straight cylindrical nanowires, where the stray field (*H* > 0.1 *M*_S_) exhibits no flux channelling (inset) and there are no antivortices present in the magnetic induction.

The formation of these asymmetric magnetic flux channels not only results in a deviation from the direct horizontal coupling of the domain walls but also induces a distinctive asymmetric structure into the magnetic induction itself. Indeed, when the magnetic induction is projected onto the *x*–*z* plane perpendicular to the direction of the domain walls (indicated in Fig. 4a), the asymmetric $\hat {\mathbf{x}}$ components of the induction caused by the flux channels result in the formation of a saddle-like structure surrounding the domain wall pair that resembles an antivortex quadrupole structure^31^ (Fig. 4b). We confirm the presence of antivortices in the magnetic induction by calculating the winding number of the normalized components of the induction in the *x*–*z* plane to be −1. These textures are of interest not only for their topological nature, but also for the type of magnetic force that could be generated. Indeed, the non-trivial in-plane structure exhibits well defined gradients in the *x*–*z* plane components of the magnetic field, offering a new route to the design of nanoscale gradients in the magnetic induction.

Due to the periodic geometry of the double helix, the in-plane antivortices are not isolated objects: the regular array of locked domain walls leads to an array of effective antivortices in the magnetic stray field (Fig. 4b). In addition, the magnetization configuration of the locked domain wall state in the chiral double helix forms an array of vortices of constant chirality in **B** (with winding number +1), which is defined by the chiral geometry. The combination of the alternating chiral vortices and antivortices in **B** is reminiscent of the cross-tie wall in planar magnetic elements^14,32^ (Fig. 4c), where the continuity of the magnetization requires the presence of a crossing of the magnetization between like-handed vortices^33^. Here, we observe this contained effective cross-tie *B* field domain wall-like structure composed of vortices in the magnetization and antivortices in the **B** field in free space.

To confirm the role of the chirality of the helix in the formation of these complex **B** field textures, we consider the equivalent domain wall configuration in a non-helical, achiral geometry composed of straight nanowires. To remove the helical geometry, we perform additional simulations after applying a coordinate transformation to the locked domain configuration, effectively unwinding the helices to form a pair of straight nanowires (Methods) and removing the influence of the three-dimensional chiral geometry of the helices and the associated curvilinear effects. Following the relaxation of the magnetization from the locked state under this new geometry (Fig. 4d), we observe no vertical component of the stray field coupling domains of the same direction in different nanowires (Fig. 4e), indicating that no flux channelling (as observed in the double helix, Fig. 3c) occurs. Due to the absence of flux channelling, no antivortex textures are observed in the stray field, confirming that the stray field textures observed surrounding the locked domain wall state are a direct consequence of the twisting of the chiral helix structure.

We have demonstrated that the three-dimensional geometry not only can alter intrastructure properties, but also offers an opportunity to tailor the magnetic field itself. This is showcased in our double-helix system, where the three-dimensional geometry results in highly stable and robust locked domain wall pairs, with prospects for robust domain wall motion and synchronous dynamics^34,35^ in three-dimensional interconnectors, key to the realization of spin logic in large-scale integrated three-dimensional circuits. These phenomena are of great interest for domain wall conduit-based information processing^36^, which includes emerging applications such as reservoir computing^2,37^ where the strong interaction between neighbouring magnetic textures and the controlled reconfigurability that these systems present is of key importance. In particular, the introduction of nonlinear interactions into a system provides the opportunity for the combination of information transmission and processing, and the possibility to go beyond von Neumann computing architectures. Moreover, the creation of an array of planar antivortices in the magnetic field in free space sets a precedent for the creation of topological magnetic field textures with complex nanoscale field gradients using three-dimensional magnetic nanostructures. The design of controlled gradients in the magnetic field is key for applications such as particle^18^ and cold-atom^17^ trapping, while the ability to define complex nanotextures in the magnetic field has important implications for imaging^19,38,39^ and magnetic field manipulation^40^. While emergent topological features in the magnetic stray field have previously been found to result in chiral behaviour in frustrated nanomagnet arrays^41^, here three-dimensional nanopatterning results in the controlled creation of well localized magnetic field antivortices. These results demonstrate that the properties of a three-dimensional system can not only be used to tailor the material internal spin states but also play a key role in defining the magnetic stray field, and thus the interaction of neighbouring features in the magnetization.

## Methods

### Three-dimensional geometrical parameters of the cobalt double helices

In curved three-dimensional systems, it is useful to define the geometrical properties in terms of the *κ* and *τ* of the system, which are defined as

$$\kappa = \frac{{r_{\mathrm{H}}}}{{r_{\mathrm{H}}^2 + P^2}},\quad\tau = \frac{{CP}}{{r_{\mathrm{H}}^2 + P^2}}$$

where *P* = *p*_H_/2π, *C* is the chirality and the radii of curvature and torsion are defined as *κ*^−1^ and *τ*^−1^, respectively. It has been shown analytically that the radii of curvature and torsion determine the effective anisotropy and spin wave dynamics of a single magnetic helix^20^. The helices investigated experimentally in this work have the geometrical parameters defined in Table 1.

### Fabrication

The three-dimensional cobalt double helices were fabricated using focused electron beam induced deposition combined with a program compatible with computer-aided design software that allows for the deposition of three-dimensional architectures. Specifically, a single-pixel double-helix model was designed using the open-source program FreeCAD. Following the growth of calibration structures to account for the growth rate of the cobalt precursor according to ref. ^23^, a stream file to direct the scanning electron beam was created.

The double helices were fabricated on Omniprobe transmission electron microscopy sample holders, which were premilled with the focused ion beam to prevent shadowing of the sample holder during the laminography scan. Both the focused ion beam milling and focused electron beam induced deposition were performed in a Helios 660 NanoLab focused ion beam microscope at the Kelvin Nanocharacterisation Centre of the University of Glasgow. Specifically, for the growth of the three-dimensional magnetic nanostructures, an acceleration voltage of 5 kV and a current of 86 pA were used, in combination with the precursor Co_2_(CO)_8_. The growth times varied between 20 and 30 min per double-helix structure.

Following the deposition, the samples were annealed for 40 min at 250 °C, which results in an increase in the spontaneous magnetization of the cobalt, without a large increase of crystalline size or texture formation^42,43^. In particular, the annealing leads to a nanocrystalline microstructure, a cobalt composition of ~80 at.% and a saturation magnetization of 800–900 kA m^−1^. This treatment also leads to the formation of a protective carbon shell around the surface of the three-dimensional structure. The annealing procedure has the additional advantage of reducing carbon deposition—and in turn any deformation of the structure—during the X-ray imaging experiments.

### X-ray magnetic laminography

Soft-X-ray magnetic laminography was performed at the PolLux beamline at the Swiss Light Source, Switzerland^13^. X-ray magnetic laminography is a recently developed three-dimensional imaging technique, which involves the measurement of projections of the magnetization of a sample for many different orientations of the sample with respect to an X-ray beam^12^.

Sensitivity to the magnetization is obtained by probing the XMCD. In this measurement, the XMCD was probed at the Co L_2_ edge with a photon energy of 796 eV. Although the XMCD contrast is weaker at the L_2_ edge than at the L_3_ edge, measurements were performed at the L_2_ edge due to the high absorption of the double helices, providing a balance between the transmission and magnetic contrast to optimize image quality. We note that, due to the higher curvature of helix A, the effective thickness of the nanowire probed by the X-rays is higher, leading to a lower signal to noise ratio in the image compared with helix B under otherwise equal imaging conditions. For each orientation, XMCD images were measured by measuring STXM images with C+, C− and linear (horizontal) polarization. To obtain a quantitative measure of the projection of the magnetization, so-called ‘dark-field’ signal originating from leakage of the centre stop of the zone plate and from the higher-order light diffracted by the monochromator was removed from the projections by applying the following normalization procedure:

$$T = \frac{{I - {\mathrm{DF}}}}{{I_0 - {\mathrm{DF}}}}$$

where *T* is the normalized transmitted intensity, *I* is the nominal transmitted intensity, *I*_0_ is the intensity incident on the sample obtained from an empty region of the image and DF is the dark-field signal, which is estimated from regions of the image where the incident beam is blocked. The linear light projections were used to cross-check the removal of the unfocused and higher-energy light. In this way, quantitative projections of the magnetization were obtained, which were used to obtain a correct reconstruction of the three-dimensional magnetization.

For the two-dimensional imaging of the helices shown in Fig. 1 before and after the application of a transverse magnetic field, the samples were mounted in the laminography stage, and aligned such that the X-rays were aligned with the direction of the long axis of the helix, resulting in an incident angle of the X-rays and long axis of the helix of 45°, providing sensitivity to the component of the magnetization parallel to the long axis of the helix. The images in Fig. 1f,h,g,i were measured with eight, two, nine and four averages, respectively, leading to slight changes in the noise level of the individual images.

For the three-dimensional imaging, the X-ray laminography set-up consisted of a rotation stage whose rotation axis is oriented at 45° to the X-ray beam. Projections were measured with an angular separation of 10°. In laminography, the number *N* of projections measured over 360° required to achieve a spatial resolution Δ*r* is defined as^44^.

$N = \uppi \frac{t}{{{\Delta}r}}\tan \theta _{\mathrm{L}}$, where *t* is the thickness of the sample and *θ*_L_ is the laminography angle, which defines the angle between the X-ray beam and the rotation axis—in this case 45°.

In this measurement, projections of the structure with a field of view of 3 × 3 μm^2^ and a pixel size of 25 nm were measured around 360° with an angular separation of 10°. For each angle, an average of two XMCD projections was measured to increase the signal to noise ratio. The angular separation of 10° corresponds to a nominal Δ*r* of less than 20 nm. In reality, the spatial resolution of the final reconstruction was limited by the signal to noise ratio of the XMCD projections. Of the 36 projections, it was only possible to measure 27 because of shadowing of the X-ray beam due to the sample holder. Simulations of magnetic laminography revealed that, although this led to an asymmetry in the reconstructed magnetization, it does not prevent the identification of the locked domain wall state.

The three-dimensional magnetic configuration was then reconstructed using a graphics processing unit implementation of an arbitrary projection reconstruction algorithm developed in ref. ^29^ and used in refs. ^12,13^.

The three-dimensional magnetization structure was visualized with ParaView 5.5.0.

### Magnetic laminography reconstruction

The reconstructed *m*_*x*_, *m*_*y*_ and *m*_*z*_ components of the magnetic configuration are presented in Extended Data Fig. 1a–c, respectively, and are directly compared with the micromagnetic simulation of the locked domain wall state in Extended Data Fig. 1d–f. The multidomain structure is confirmed by the *m*_*x*_ component, which reveals positive and negative domains on the upper and lower halves of the helix, respectively. However, it is only with the transverse components of the magnetization, *m*_*y*_ and *m*_*z*_, that the type of domain wall pair state can be identified: both the *m*_*y*_ and *m*_*z*_ components exhibit an alternating contrast (Extended Data Fig. 1b,c), consistent with the locked domain wall state (Extended Data Fig. 1e,f), and not with the more standard unlocked domain wall pair configuration previously observed in planar systems.

### Micromagnetic simulations

Finite-element meshes were created by first creating STL files corresponding to three-dimensional double helices of different parameters using the open-source program FreeCAD. For all simulations, the nanowire diameter was kept constant at 50 nm, while the helix radii and pitches were varied. The meshes were created with a mesh size of 5 nm using the program Gmsh^45^.

To map the phase diagram of the stable state after transverse saturation of the magnetization, micromagnetic simulations were performed using the program magnum.fe, which employs finite-element micromagnetics with a hybrid finite-element/boundary-element method for the magnetostatic field computation and a preconditioned implicit time integration scheme for the Landau–Lifshitz–Gilbert equation^46^. The spontaneous magnetization of the material was fixed to *M*_s_ = 8 × 10^5^ A m^−1^. The magnetization was initialized in the transverse direction, assuming full saturation, and then the relaxation calculated using the Landau–Lifshitz–Gilbert equation with a damping parameter *α* = 1.

To calculate the stray field around the final magnetization state, the double-helix mesh was embedded within a boxed mesh using the program Gmsh, and the stray field calculated at each position using the magnum.fe solver.

To compare the helix configuration with that of a straight nanowire, a coordinate transformation was applied to the relaxed locked domain wall configuration result using ParaView, and the magnetization transformed accordingly. Following the unwinding of the magnetic state, the configuration was once more allowed to relax using magnum.fe to reach a stable configuration. The micromagnetic simulations presented in Figs. 3 and 4 of the main text correspond to a helix pitch of 500 nm, a helix radius of 30 nm and a nanowire diameter of 50 nm.

## Online content

Any methods, additional references, Nature Research reporting summaries, source data, extended data, supplementary information, acknowledgements, peer review information; details of author contributions and competing interests; and statements of data and code availability are available at 10.1038/s41565-021-01027-7.

## Supplementary information

Supplementary Information Supplementary Discussion and Figs. 1–4.

Supplementary Data 1 Source Data Supplementary Fig. 3. The source data for the phase diagrams are given as txt files.

Supplementary Data 2 Source Data Supplementary Fig. 4. The source data for the two phase diagrams, for the case of nanowire radius 25 nm (orange) and 40 nm (purple) are given as txt files.

## Data Availability

All data associated with this manuscript are available on the Zenodo repository at 10.5281/zenodo.5657298.

## Code availability

All analysis code and reconstruction algorithms associated with the work of this manuscript are available on the Zenodo repository at 10.5281/zenodo.5657298.
