# Supplementary material for: Complex free-space magnetic field textures induced by three-dimensional magnetic nanostructures
Source: Nat Nanotechnol. 2021 Dec 20;17(2):136–42. doi: 10.1038/s41565-021-01027-7 (PMC8850196; doi:10.1038/s41565-021-01027-7)
Supplement: Supplementary file 1 — Supplementary Discussion and Figs. 1–4. [file 41565_2021_1027_MOESM1_ESM.pdf]

nature portfolio

https://doi.org/10.1038/s41565-021-01027-7

Supplementary information

Complex free-space magnetic field textures induced by three-dimensional magnetic nanostructures

In the format provided by the authors and unedited

Complex free-space magnetic field textures induced by 3D magnetic nanostructures

Author List:

Claire Donnelly[1,2*], Aurelio Hierro-Rodríguez[3,4,5], Claas Abert[6], Katharina Witte[7,8], Luka Skoric[1], Dédalo Sanz-Hernández[1], Simone Finizio[7], Fanfan Meng[1], Stephen McVitie[3], Jörg Raabe[7], Dieter Suess[6], Russell Cowburn[1], Amalio Fernández-Pacheco[9*]

Affiliations:

[1] Cavendish Laboratory, University of Cambridge, JJ Thomson Ave, Cambridge, CB3 0HE, UK.

[2] Max Planck Institute for Chemical Physics of Solids, 01187 Dresden, Germany.

[3] SUPA, School of Physics and Astronomy, University of Glasgow, Glasgow G12 8QQ, UK.

[4] Departamento de Física, Universidad de Oviedo, 33007 Oviedo, Spain.

[5] CINN (CSIC-Universidad de Oviedo), 33940 El Entrego, Spain.

[6] Faculty of Physics, University of Vienna, 1090 Vienna, Austria.

[7] Swiss Light Source, Paul Scherrer Institute, 5232 Villigen, Switzerland.

[8] Berlin Partner für Wirtschaft und Technologie GmbH, 10623 Berlin, Germany

[9] Instituto de Nanosciencia y Materiales de Aragón, 50009 Zaragoza, Spain.

*Correspondence to: claire.donnelly@cpfs.mpg.de, amaliofp@unizar.es

Supporting Information:

I) Phase Diagram

To understand the influence of the geometry on the formation of the different remanent states – the locked, unlocked domain walls, and the antiparallel state (**Supplementary Figure 1a**) – we map the phase diagram of the relaxed magnetic configurations for a variety of geometries, by varying the pitch and radius of the double helix geometry (**Supplementary Figure 1b**). The locked domain wall pairs occur in a red "pocket" corresponding to higher helix pitch and lower helix radius – or, equivalently, higher torsion and lower curvature. We can understand this phase diagram by considering the influence of the geometry on the competing magnetic interactions. As the radius and the curvature of the helix increases, the curvature-induced anisotropy increases, while the inter-wire distance also increases, lowering the magnetostatic coupling of a domain wall pair. In regions of lower curvature and lower helix radius, the domain walls therefore reorient after transverse saturation. This is due to the magnetostatic interaction overcoming the curvature-induced magnetic anisotropy of the domain wall, to close the magnetic flux and reduce the magnetostatic energy, as shown schematically in **Figure 2b**. This contrasts with helices with a lower pitch, where the inter-domain wall distance within a single helix is reduced, favouring the annihilation of the domain wall pairs and the formation of the antiparallel state. We reproduce this phase diagram with a simple analytical model (see next section of Methods), confirming that the locked domain wall state forms as a direct result of the fine balance of these competing effects.

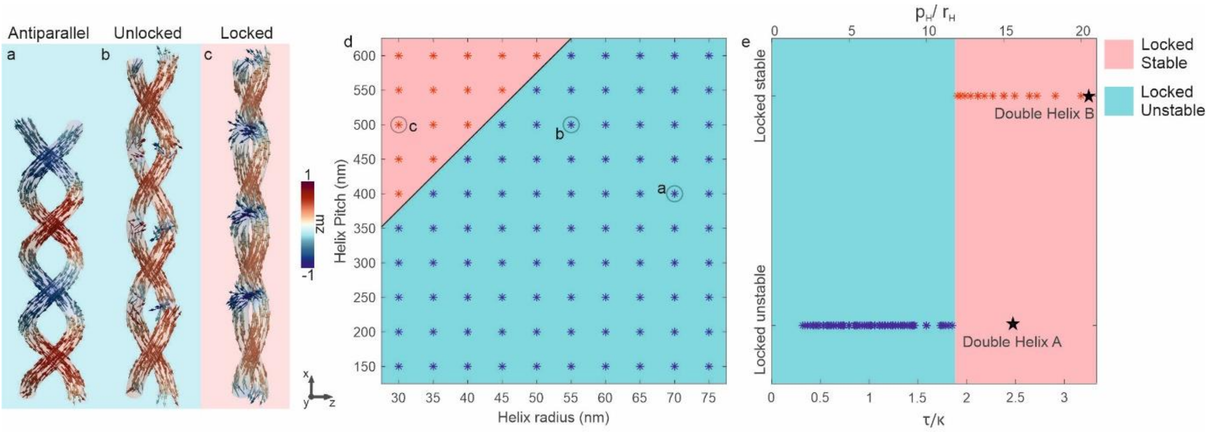

Supplementary Figure 1. Finite element micromagnetic simulations of double helices with varying pitch and radii, revealing three stable configurations after the presence of a transverse saturating magnetic field: a) the antiparallel state, as well as regularly spaced c) locked and b) unlocked domain wall pairs. d) A phase diagram of the states following saturation reveals that the locked domain wall state occurs in an isolated pocket (pink) corresponding to high helix pitch and small helix radius. e) The locked domain wall state is stable for pitch/radius ratios above a threshold value, which corresponds to a high torsion: curvature ratio. The ratios of the experimentally investigated helices are plotted as a reference.

We gain further insight into the stability of the locked domain wall state by replotting the diagram as a function of the helix torsion:curvature ratio (τ/κ) in **Supplementary Figure 1c**, which reveals that the locked state is stable for τ/κ ratios above 1.9 for this material parameters. This transition in the τ/κ ratio demonstrates that higher τ and lower κ promote the increase of the inter-domain wall coupling, overcoming the curvature-induced anisotropy to form tightly bound domain wall pairs. Indeed, the τ/κ ratio of the cobalt double helices (**Supplementary Figure 1c**) mirrors this trend, with Double Helix B, which exhibits a stable domain wall state, having an increased τ/κ ratio. We note that both experimental states of Double Helices A and B are located in the "locked" region computed in the simulations, with Double Helix A closer to the regime where the locked state becomes unstable. We associate this quantitative discrepancy for Double Helix A, where an antiparallel state is always observed in experiments and thus the locked state is not experimentally stable, to the slightly larger nanowire diameters of the experimental Double Helices A and B compared to the simulations, which results in a shift of the threshold to higher τ/κ. Indeed, our analytical model confirms this (see next section of Methods).

II) Analytical model reproducing the phase diagram:

With micromagnetic simulations, we determined that the stability of the locked domain wall state is highly influenced by the geometry of the double helix system – and the relative curvature and torsion, as shown in **Supplementary Figure 2**. In order to elucidate the physics behind this influence, we developed a simple quasi-1D analytical model of the double helix structure.

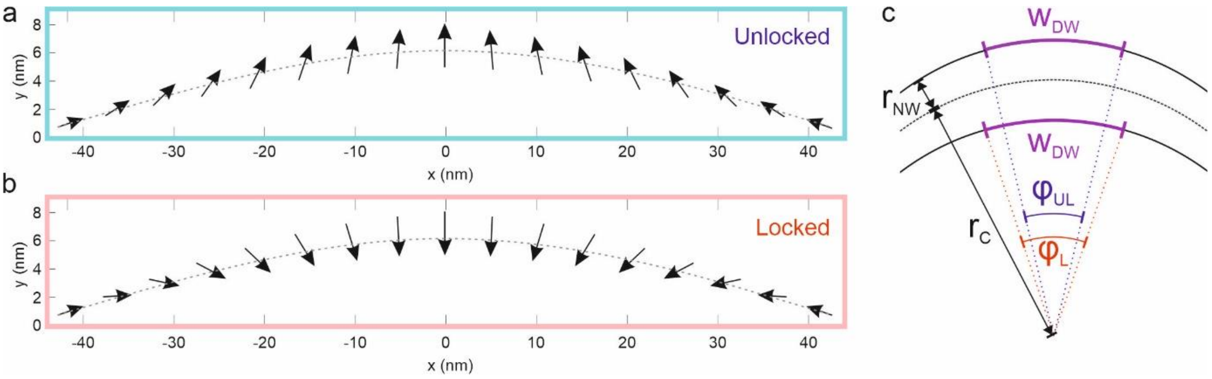

Supplementary Figure 2. Schematic of analytical model of the unlocked (a) and locked (b) domain wall configurations which are show a positive and negative rotation of the moment across the domain wall width, respectively. c) The different

distribution of magnetic charges on the surface of the nanowire for the two states leads to a different angle spanned by the domain wall φ.

Specifically, we model each nanowire in the double helix nanostructure as a helix of exchange coupled magnetic moments and determined the influence of the helix parameters on the exchange energy and magnetostatic energy of the domain walls in the system.

We first define the "locked" and "unlocked" domain wall states mathematically by assuming a continuous rotation of the magnetisation from the direction tangential to the nanowire via a positive or negative direction across the width of the domain wall, as shown schematically in **Supplementary Figure 2**.

We determine the domain wall width by conserving the flux of the magnetisation, assuming that the net charge of the domain wall is distributed over an azimuthal angle of $\frac{\pi}{3}$, and that this is opposite and equal in magnitude to the charge distributed over the cross section of the domains in the nanowire:

$$2\pi {r_{NW}}^2 = w_{DW}\theta r_{NW}$$

Where r_{NW} is the radius of the nanowire, θ is the azimuthal angle over which the charges are distributed (in this case assumed to be $\frac{\pi}{3}$) and w_{DW} is the width of the domain wall (on the surface of the nanowire). As the net surface charges are distributed on the inner and outer surfaces of the curved helix for the locked and unlocked domain wall, respectively, this translates to a difference in the angle spanned by the domain wall for the two different states as shown in **Supplementary Figure 2c** and giving:

$$\varphi_{UL} = \frac{w_{DW}}{(r_C + r_{NW})}, \qquad \varphi_L = \frac{w_{DW}}{(r_C - r_{NW})}$$

Where r_C is the radius of curvature of the helices. Based on this model, we find that the domain wall in the locked domain wall state thus spans a larger angle than that of the unlocked state. By calculating the differences in the exchange energy, the "self" magnetostatic energy, and the "interaction" magnetostatic energy for the locked and unlocked domain wall state, we can identify a region of the phase diagram for which the locked domain wall state is stable, as seen in **Supplementary Figure 3a**. In this phase diagram, we see that the locked domain wall state is stable for regions of lower helix radius, mainly due to the magnetostatic interaction of the domain walls, as described previously.

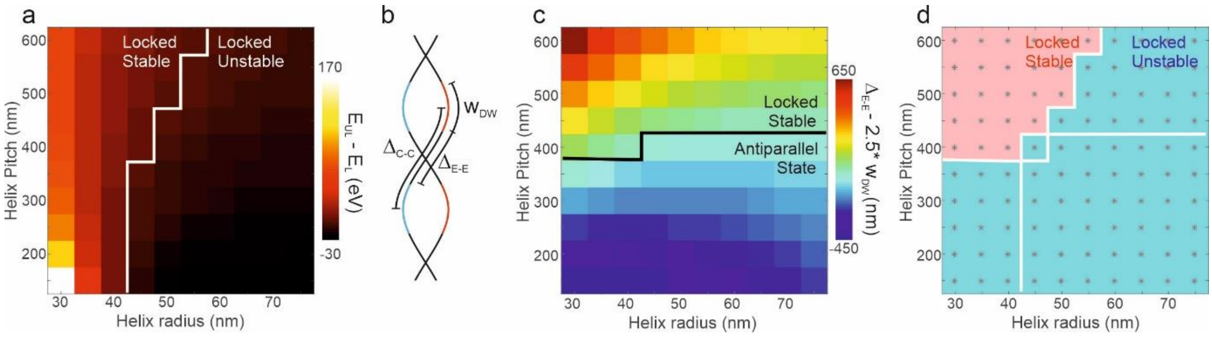

Supplementary Figure 3. Outcome of analytical model determining the stability of the locked domain wall state. a) the combination of exchange and magnetostatic energy leads to the locked domain wall state being more stable for helices with lower pitch. b) By considering the distance between domain wall pairs that form in the double helix structure, the likelihood of the domain walls to annihilate is estimated c) revealing that the locked domain wall state is stable for higher helix pitches. d) Combining the two, the pocket corresponding to the stable locked domain wall state seen with micromagnetic simulations is recovered.

We next consider a second effect, which is the stability of the locked domain wall state not with respect to the unlocked domain wall state, but with respect to the likelihood of domain wall annihilation. Indeed, it is through the annihilation of neighbouring domain wall pairs that leads to the antiparallel state, as

observed with micromagnetic simulations for double helices with lower pitch. We model the likelihood of the domain walls annihilating by calculating the distance between neighbouring domain wall pairs in the helix (shown in **Supplementary Figure 3b**) and comparing this to the width of the domain wall. By defining a threshold distance below which the domain walls are likely to annihilate, we find that the locked domain wall state is stable in the upper half of the phase diagram, corresponding to double helices with higher pitch. Combining the two effects: the total energy of the system, and the domain wall separation, we are able to reproduce the "pocket" in the phase diagram corresponding to high helix pitch and low helix radius, as seen with the micromagnetic simulations (**Supplementary Figure 1b**).

When comparing the micromagnetic simulations with the experimental data, and specifically the stable state as a function of the torsion/ curvature ratio in **Supplementary Figure 1c**, it was observed that both Double Helix A and Double Helix B lie within the region corresponding to a stable locked domain wall state. This discrepancy can be understood when we take into consideration that the radius of the nanowire in the experiments is on the order of 40 nm, while the radius of the nanowire in the micromagnetic simulations is lower at 25 nm. Indeed, when we model the phase diagram analytically for the two nanowire radii, we observe a shift in both the pocket corresponding to the stable locked domain wall state (**Supplementary Figure 4b**) as well as a shift in the torsion:curvature threshold to higher values (**Supplementary Figure 4c**), accounting for the experimental results.

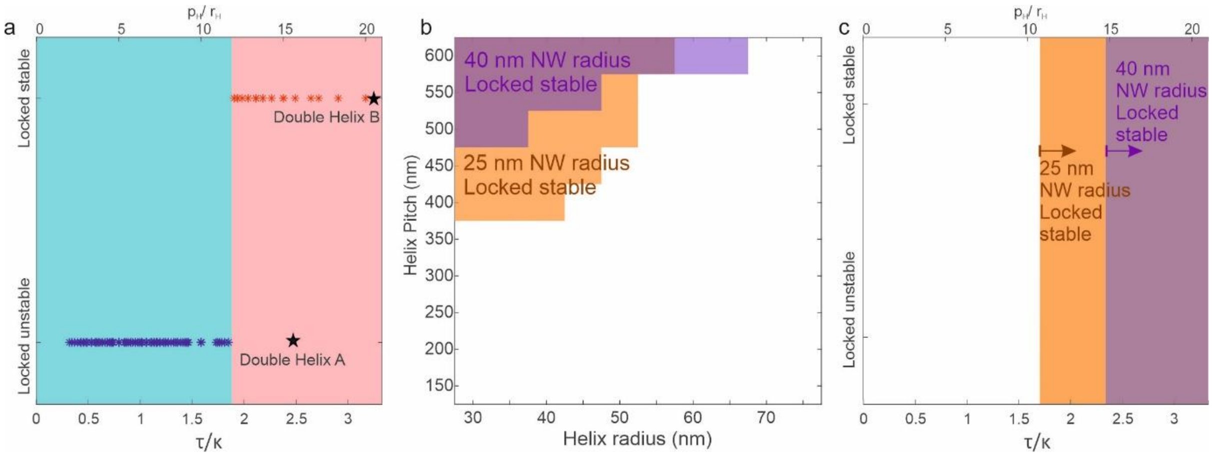

Supplementary Figure 4. The effect of the nanowire diameter on the phase diagram of the stability of the locked domain wall state to explain the discrepancy between the experimental data and micromagnetic simulations in a). Specifically, an increase of the nanowire radius from 25 nm to 40 nm leads to the pocket of locked domain wall stability moving to higher helix pitch (b) and a shift of the threshold in the torsion:curvature ratio to higher values, accounting for the locked domain wall state not being stable in Double Helix A.

III) Discussion of curvature-induced effects

Curvature and torsion result in two main effects: the curvature-induced anisotropy, and the curvature-induced DMI [1], which occur due to a combination of magnetostatic and exchange interactions. The curvature-induced anisotropy can be defined as:

$$K_1 = 1 + \sigma^2 - \chi^2 \ \text{ and } \ K_2 = \chi^2$$

where $\boldsymbol{K_1}$ and $\boldsymbol{K_2}$ are the anisotropy constants parallel and perpendicular to the tangent of the helix, respectively, defined in terms of the reduced torsion $\boldsymbol{\sigma = \tau l}$, the reduced curvature $\boldsymbol{\chi = \kappa l}$, where $\boldsymbol{l = \sqrt{A/K^{eff}}}$ is the characteristic magnetic length (exchange length).

The equivalent expressions for the curvature-induced DMI terms are as follows:

$$D_1 = 2\sigma \text{ and } D_2 = 2\chi$$

For our double helices, we can calculate both the effective anisotropy and DMI terms, based on the quantitative values of the curvature and torsion:

$l = 5e-9$	$\boldsymbol{\kappa}$ (nm^{-1})	$\boldsymbol{\tau}$ (nm^{-1})	$\boldsymbol{\chi = \kappa l}$	$\boldsymbol{\sigma = \tau l}$	$\boldsymbol{K_1}$	$\boldsymbol{K_2}$	$\boldsymbol{D_1}$	$\boldsymbol{D_2}$
Helix A	1.91e-3	4.76e-3	9.55e-3	0.0238	1.00048	9.1e-5	0.0474	1.91e-2
Helix B	1.31e-3	4.26e-3	6.55e-3	0.0213	1.00041	4.3e-5	0.0426	1.31e-2

In a single domain helix, the curvature and torsion result in a tilting of the magnetisation away from the tangent to the helix by an angle:

$$\varphi = \sigma\chi$$

For Helix A and B, the curvature and torsion result in a tilt of the equilibrium magnetisation from the tangential direction by an angle of 0.01° and 0.008°, respectively, in agreement with the quasi-tangential state observed in the XMCD projections.

We next consider the domain wall states where the curvature leads to an effective DMI, promoting the unlocked domain wall state over the locked domain wall state, that is overcome by the inter-helix interaction. We note that due to the small magnitude of the curvature in our experimental systems, the curvature-induced effects on the domain walls are dominated by the magnetostatic interaction [2]. Indeed, in the micromagnetic simulations we observe that the relaxation of the magnetisation from the unlocked (curvature preferred) into the locked (curvature unpreferred) state is associated with a significant increase in the internal magnetostatic energy of the helices, and a small decrease in the exchange energy due to a change in the domain wall width. This indicates that in three-dimensional systems, with a nanowire diameter of 70-80 nm, the behaviour is not dominated by exchange but rather magnetostatics, resulting in the exchange-driven curvature effects described in the literature not being observed.

[1] Pylypovskyi, O. *et al.*, *Rashba Torque Driven Domain Wall Motion in Magnetic Helices*. Scientific Reports **6**, 23316 (2016).

[2] Volkov, O.M., *et al.*, *Experimental Observation of Exchange-Driven Chiral Effects in Curvilinear Magnetism*. Physical Review Letters **123**, 077201 (2019).
